# Emergence and genomic characterization of *Proteus mirabilis* harboring *bla*_NDM-1_ in Korean companion dogs

**DOI:** 10.1186/s13567-024-01306-w

**Published:** 2024-04-09

**Authors:** Su Min Kyung, Jun Ho Lee, Eun-Seo Lee, Xi-Rui Xiang, Han Sang Yoo

**Affiliations:** Department of Infectious Disease, College of Veterinary Medicine, Seoul National University, Seoul, Republic of Korea

**Keywords:** Carbapenem, carbapenemase, multidrug resistance, NDM, *Proteus mirabilis*, whole-genome sequencing, epidemiology, One health

## Abstract

*Proteus mirabilis* is a commensal bacterium dwelling in the gastrointestinal (GI) tract of humans and animals. Although New Delhi metallo-β-lactamase 1 (NDM-1) producing *P. mirabilis* is emerging as a threat, its epidemiology in our society remains largely unknown. LHPm1, the first *P. mirabilis* isolate harboring NDM-1, was detected from a companion dog that resides with a human owner. The whole-genome study revealed 20 different antimicrobial resistance (AMR) genes against various classes of antimicrobial agents, which corresponded to the MIC results. Genomic regions, including MDR genes, were identified with multiple variations and visualized in a comparative manner. In the whole-genome epidemiological analysis, multiple phylogroups were identified, revealing the genetic relationship of LHPm1 with other *P. mirabilis* strains carrying various AMR genes. These genetic findings offer comprehensive insights into NDM-1-producing *P. mirabilis*, underscoring the need for urgent control measures and surveillance programs using a “one health approach”.

**Supplementary Information:**

The online version contains supplementary material available at 10.1186/s13567-024-01306-w.

## Introduction

*Proteus mirabilis* is a ubiquitous bacterium found not only in soil, water, and sewage environments but also as a commensal bacterium in the gastrointestinal (GI) tract of humans and animals [1]. *P. mirabilis* is one of the most significant pathogens responsible for urinary tract infections (UTIs) in health care settings, with a suspected origin of the GI tract [1, 2]. *P. mirabilis* is also responsible for various clinical conditions, including respiratory tract infections, neonatal meningoencephalitis, empyema, and osteomyelitis [3]. Several broad-spectrum antibiotic agents, such as ampicillin, amoxicillin-clavulanate, ceftriaxone, ciprofloxacin, levofloxacin, trimethoprim-sulfamethoxazole (TMP-SMX), piperacillin-tazobactam, and carbapenems, are important treatment options for clinical *P. mirabilis* infections. Therefore, the emergence of carbapenemase-producing *P. mirabilis* could dramatically reduce treatment options for these clinical infections [4].

Multidrug-resistant (MDR) bacterial infection, especially that caused by New Delhi metallo-β-lactamase 1 (NDM-1)-producing gram-negative bacteria has already become a major threat to the nosocomial environment and public health [5–7]. Carbapenemase-producing strains acquire resistance to more than 2–3 classes of antibiotics, including carbapenems [8], which makes it incredibly difficult to treat these MDR pathogens in clinical situations. With a major association with mobile gene elements such as conjugative plasmids and integrative conjugative elements (ICEs) [9–11], *bla*_*NDM-1*_ is rapidly mutating and disseminating worldwide.

Carbapenem usage in animals is not allowed globally, yet unauthorized adjustments of the drugs in animal clinical environments remain, leading to the dissemination of carbapenemase-producing strains as an unaddressed threat. Therefore, the unidentified dissemination of carbapenemase producers among companion animals in our society should be considered a major threat to public health [12, 13]. Carbapenemase-producing bacteria are not a concern limited to human health; they require novel and urgent control measures and should not be neglected. However, there is only a limited amount of investigative data on carbapenemase-producing strains in animals in our society.

In this study, we employed molecular epidemiological approaches to investigate a carbapenemase producing *P. mirabilis* isolate. Because this was the first identification of an NDM-1 harboring *P. mirabilis* isolated from a companion dog in South Korea, a molecular approach in a whole-genome manner was performed to obtain detailed information about the genomic characteristics of the identified strain. The aim of our study was to gain a comprehensive understanding of future control measures in order to achieve the “one health approach” in our society.

## Materials and methods

### Bacterial strain isolation, carbapenemase gene detection and minimum inhibitory concentration determination

Swabbed samples obtained from rectal and nasal swabs of dogs and cats visiting an animal clinical hospital in Seoul, South Korea, were subjected to screening on meropenem-impregnated (1 μg/mL) MacConkey (MIM) agar for carbapenem-resistant strain identification. The total bacterial DNA was isolated using the Wizard Genomic DNA purification kit (Promega, Madison, WI, USA). For the identification of carbapenemase genes, PCR screening using previously described primers [14] was initially performed, and the confirmed strains were subjected to minimum inhibitory concentration (MIC) level identification and next-generation sequencing (NGS). The identified microbial species were determined with matrix-assisted laser desorption ionization–time of flight-mass spectrometry (MALDI-TOF-MS; Bruker Daltonik GmbH, Bremen, Germany).

The MICs of the isolates were determined for 14 antimicrobial agents using E-test (Biomerieux, Marcy L’Étoile, France) technique in accordance with the manufacturer’s instructions. *E. coli* strain ATCC 25922 was used as a quality control strain and only drugs with quality standards were used in the test.

### Illumina/MinION sequencing and characterization

For whole genome sequencing, purified DNA acquired from a Wizard Genomic DNA Purification Kit (Promega, Madison, WI, USA) was used. In summary, two independent genomic DNA libraries were prepared for both short and long read systems and sequenced. Long read genomic sequencing with Oxford Nanopore (Oxford Nanopore Technologies, Oxford, UK) platforms was corrected using Illumina NovaSeq 6000 (Illumina, San Diego, CA, USA) following a paired-end 2 × 150-bp protocol.

The DNA library was prepared according to the Illumina TruSeq DNA PCR-Free Library Prep protocol (Cat. 20015963). For sample library preparation, 2 μg of 550 bp inserts of high molecular weight genomic DNA was randomly sheared to yield DNA fragments using the Covaris S2 system. The fragments were blunt ended and phosphorylated, and a single “A” nucleotide was added to the 3’ ends of the fragments in preparation for ligation to an adapter that has a single-base “T” overhang. Adapter ligation at both ends of the genomic DNA fragment conferred different sequences at the 5’ and 3’ ends of each strand in the genomic fragment. The quality of the libraries was verified by capillary electrophoresis (Bioanalyzer, Agilent).

The ONT library was constructed by using a Ligation Sequencing Kit (SQK-LSK109). This library was sequenced with a Ligation Sequencing Kit (SQK-LSK109), a Flow Cell Priming Kit (EXP-FLP002), and a Flowcell (FLO-MIN106). All runs were performed on the MinION sequencer.

After quantification using the QX200 Droplet Digital PCR System (Bio-Rad), we combined libraries that were index tagged in equimolar amounts in the pool. WGS sequencing was performed using the Illumina NovaSeq 6000 system according to the protocol provided for 2 × 150 sequencing.

Reads were trimmed and filtered for long and high-quality reads using FiltLong v0.2.0 [15]. Filtered long read data were prepared using Flye v2.8.3 to proceed de novo assembly [16] and checked to determine whether they were circular or linear for the contig produced as a result of assembly using Circlator v1.5.5 [17]. Data polishing was carried out with Pilon v1.23 [18] for the contigs whose structural shape was identified, and evaluation of the assembly results was carried out through BUSCO v4.1.2 [19]. After polishing, structural annotation was performed using Prokka v1.14.6 [20] for the contigs to determine the location, length and number of CDS, rRNA and tRNA. Functional annotation was performed with DIAMOND v 0.9.26 [21] to process the file, and then Blast2GO v4.1.9 [22] was used to perform Gene Ontology analysis.

### Genetic structure analysis and bioinformatic comparison

For genomic structure visualization of the whole chromosome, CGView [23] was employed. Easyfig 2.2.3 was used for the pairwise BLASTn alignment of genomic structures, including characteristic transposons.

A total of 98 whole-genome datasets of *P. mirabilis* strains were downloaded from the National Center for Biotechnology Information [24] for comparison with the sequence in this study by bioinformatic manners. The assembled genomes were screened for comparison of the resistance genes and plasmid types on the Center for Genomic Epidemiology (CGE) server [25] in silico utilizing ResFinder 4.1 and PlasmidFinder 2.1. The whole-chromosome structure map was generated using the CGView server [23], with *P. mirabilis* HI4320 (GenBank accession no. AM942759.1) applied as a backbone. Whole-chromosome single nucleotide polymorphism (SNP) datsets were generated [26], and a concatenated alignment was created using CSI Phylogeny [27] with standard settings and using *P. mirabilis* HI4320 (GenBank accession no. AM942759.1) as a reference. MEGA 11 software [28] was employed for reliable maximum likelihood (ML) tree construction with 1000 bootstrap replicates. The epidemiological visualization was generated on iTOLs [29] for comparison of the characteristic information heatmaps along with the whole-genome phylogenetic tree.

This study describes the first carbapenemase-producing *P. mirabilis* strain isolated from a companion animal in South Korea. The identified whole-genome structure revealed 20 different AMR genes, including 2 tandem copies of *bla*_*NDM-1*_, which contributed to the MDR capacity of the isolate. Multiple variations in MDR gene regions and phylogenetic relatedness were identified by whole-genome analysis. The findings from the carbapenemase-producing *P. mirabilis* strain from a companion animal indicate that the problem of AMR is not limited to human health and should be addressed from the perspective of the “one health approach”.

## Results

### Characterization of carbapenemase-producing *P. mirabilis* and MIC determination

*Proteus mirabilis* strain LHPm1 was isolated on 9 April 2021 from a companion dog at a clinical animal hospital in Seoul, South Korea. The isolate was identified from a rectal swab of 6 year-old neutralized female poodle, without any characteristic clinical symptoms. Rectal swabs were performed along with nasal swabs for the purpose of screening and identifying carbapenem-resistant Enterobacterales (CRE). LHPm1 was isolated as a result of rectal swab screening on meropenem impregnated (1 µg/mL) MacConkey (MIM) agar.

As a result of PCR identification, the carbapenem-resistant isolate was found to carry the carbapenemase-producing gene *bla*_*NDM*_. Subsequently, the isolate underwent MIC evaluation and whole-genome sequencing. The MIC was evaluated against 14 different antimicrobial agents (Table 1) and was confirmed to have high (MIC value higher than 32 µg/mL) meropenem resistance. The isolate was found to have resistance against β-lactam agents, such as amoxicillin/clavulanic acid (penicillin), ceftazidime and ceftriaxone (3^rd^ generation cephalosporin), ceftolozane/tazobactam (4^th^ generation cephalosporin), and piperacillin/tazobactam (ureidopenicillin). Tetracycline, chloramphenicol, gentamicin, and polymyxins were also found to be unreliable options. Reliable options against LHPm1 included aztreonam (monobactam), amikacin (aminoglycoside), tigecycline (glycylcyclines), and nalidixic acid (quinolone).

**Table 1:** **Minimum inhibitory concentrations of 14 antimicrobial agents (µg/mL) against LHPm1**

Antimicrobial agents	MIC results (µg/mL)	Resistance determinant
Amikacin	12	S
Amoxicillin/clavulanic acid (2:1)	48	R
Aztreonam	< 0.016	S
Ceftazidime	> 256	R
Ceftriaxone	> 256	R
Ceftolozane/tazobactam	> 256	R
Chloramphenicol	96	R
Colistin	> 256	R
Doxycycline	32	R
Gentamicin	16	R
Meropenem	> 32	R
Nalidixic acid	3	S
Piperacillin/tazobactam	> 256	R
Tigecycline	0.25	

The minimum inhibitory concentration was determined with the E-test strip technique for 14 different antimicrobial agents. *E. coli* strain ATCC 25922 was used as a quality control strain.

### Sequenced and assembled whole-genome datasets

As a result of the whole genome sequencing and assembly, a high-quality sequence was generated (Additional file 1). A 4000428 base-pair long chromosome was identified, without any identifiable plasmid (Table 2).

**Table 2:** **Genomes identified as a result of sequencing and assembly**

Bacterial Strain	Gene type	Gene Length (bp)	Gene form	GC contents	CDS	rRNA	tRNA
LHPm1	Chromosome	4000428	Circular	39.17	3,579	22	84

LHPm1 was found to carry a 4 000 428 base-pair-long chromosome. A plasmid was not found in the bacterial genome.

### Whole-genome structure and characteristic gene visualization

**Figure 1:**
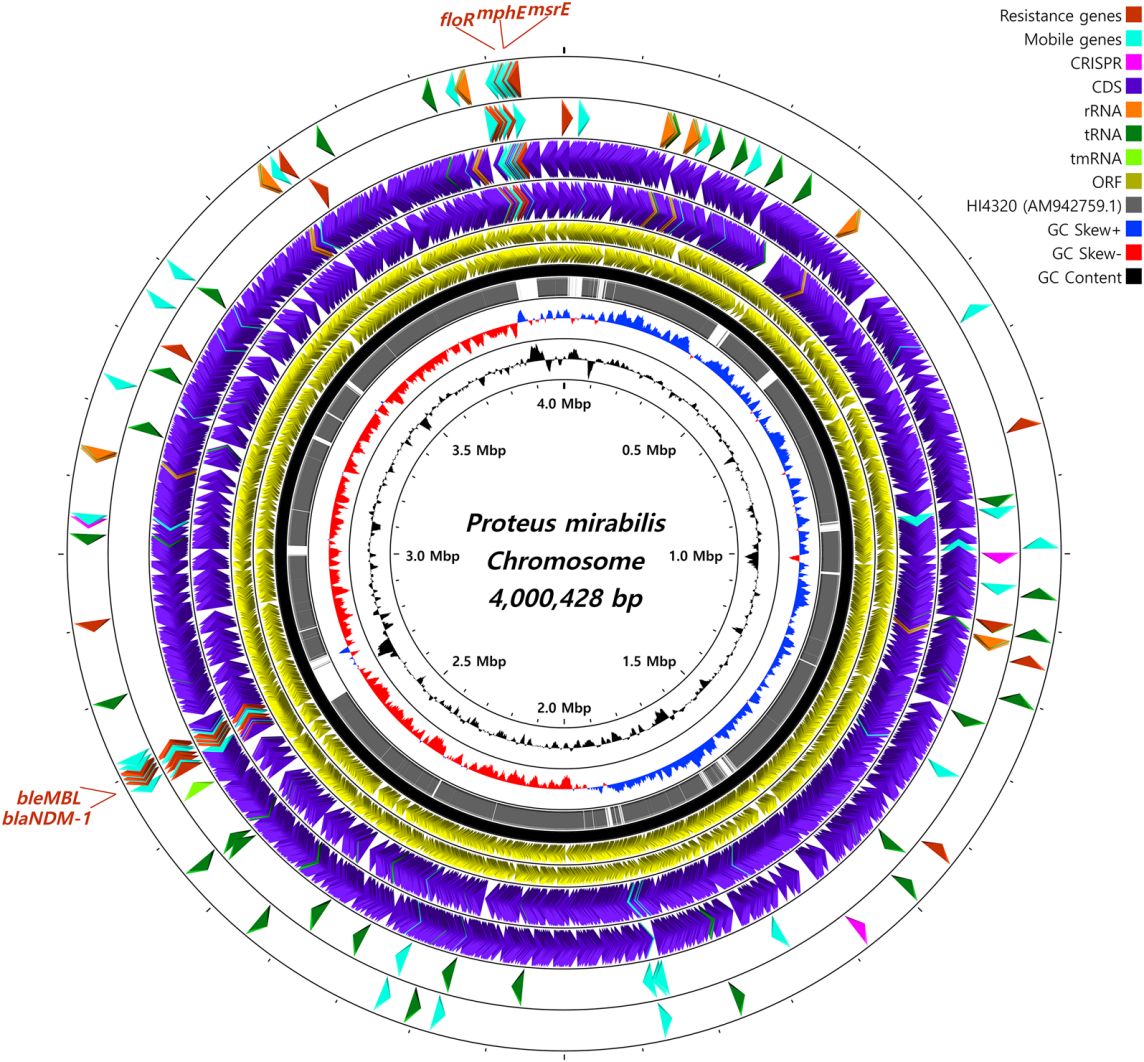
**Identification of the whole chromosome structure of LHPm1.** The whole chromosome of NDM-1-carrying *P. mirabilis* (4 000 428 bp) was identified in this study and visualized. The chromosomal map of *P. mirabilis* HI4320 (4 063 606 bp, GenBank accession no. AM942759.1) was utilized as the backbone for visualization and is depicted as a black circle. The characteristic gene positions, such as that of resistance genes and mobile gene elements, are additionally highlighted in the outermost 2 circles. The circular map was generated by CGView.

The whole-genome structure was visualized (Figure 1) using CGView, with the *P. mirabilis* HI4320 (GenBank accession no. AM942759.1) sequence as a reference. A total of 20 different antimicrobial resistance (AMR) genes were identified from the whole genome of LHPm1. Genomic positions of characteristic genes, such as AMR genes, mobile genes and CDS, are depicted with distinguishable colored arrows in the whole chromosomal map.

**Figure 2:**
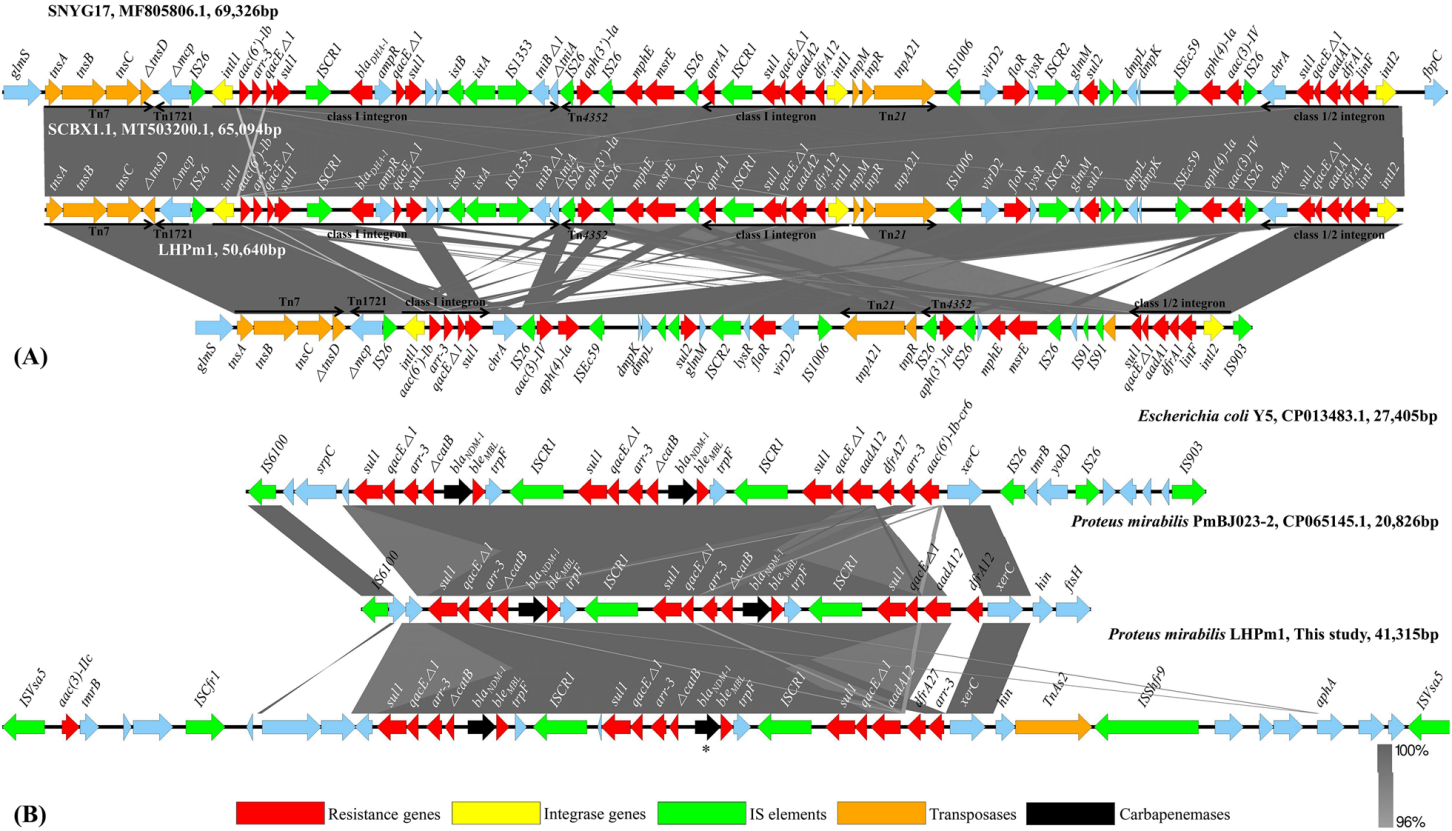
**Genomic comparison of the identified novel variants in the MDR region.** Multiple AMR genes and mobile genes were found to be concentrated in particular gene regions and visualized via linear comparison. The transposable elements of LHPm1 were identified and compared with the Tn6450 part discovered in *P. mirabilis* strain SNYG17 (GenBank accession no. MF805806.1) and the Tn6765 part in strain SCBX 1.1 (GenBank accession no. MT503200.1). Transposons and integron structures are indicated with black arrowheads. Two sites of *bla*_*NDM-1*_ were identified from LHPm1 in a variated form and comparatively visualized with corresponding structures identified from *E. coli* strain Y5 (GenBank accession no. CP013483.1) and *P. mirabilis* strain PMBJ023-2 (GenBank accession no. CP065145.1). Gray shading was adjusted to indicate corresponding shared regions with more than 96% gene identity. Easyfig 2.2.3 was used for the pairwise BLASTn alignment comparison.

Two genomic regions were identified with concentrated AMR genes and mobile genes. Comparative visualization (Figure 2) revealed the variated structure of the genomic regions, from previously reported datasets.

A 50,640-base pair-long Tn*7*-like transposon structure with a transposition module of *tnsA-tnsB-tnsC-△tnsD* was identified, with novel variations (Figure 2A). The MDR gene region was found to harbor various classes of AMR genes, namely, aminoglycoside (*aac(6’)-lb*, *aac(3)-IV*, *aph(4)-la*, *aph(3’)-la* and *aadA1*), rifampin (*arr-3*), sulfonamide (*sul1* and *sul2*), florfenicol and chloramphenicol (*floR*), macrolide (*mphE* and *msrE*), trimethoprim (*dfrA1*) and lincosamide (*linF*) AMR genes. The novel variation of the Tn*7*-like region was identified in comparison to Tn6450 (GenBank accession no. MF805806.1) and Tn6765 (GenBank accession no. MT503200.1). In particular, a class I integron was identified to be interrupted with a reversed gene region of Tn*21*, consequently leading to the deletion of the *bla*_*DHA-1*_ gene.

Two sites of the *bla*_NDM-1_ gene were identified in LHPm1 and associated with multiple mobile gene elements (Figure 2B). Both *bla*_NDM-1_ genes had base-pair substitution sites at C621A. A particular *bla*_NDM-1_ gene sequence contained an additional mutated 107T insertion and G108A substitution, speculatively resulting in a frame shift of the amino acid sequence and marked (*) in Figure 2B. The *bla*_NDM-1_ gene was coupled with mobile genes and multiple AMR genes, such as *sul1*, *arr-3*, *aadA12* and *dfrA27*. The 41,315-base pair-long gene structure between the two sites of IS*Vsa5* was different from previously reported gene structures and was comparatively visualized with corresponding similar genes identified in *E. coli* strain Y5 (GenBank accession no. CP013483.1) and *P. mirabilis* strain PMBJ023-2 (GenBank accession no. CP065145.1). While the MDR regions, including *sul1*, *arr-3*, *ble*_*MBL*_ and *bla*_*NDM-1*_, were similar, the outer structure was different among the strains.

### Epidemiologic gene characterization in comparison with worldwide datasets

A total of 98 *P. mirabilis* whole-genome datasets available from the National Center for Biotechnology Information [24] were employed for whole-genome comparison (Additional file 2). The strains were isolated between 1933 and 2021 and from 20 different countries (66 strains from China). The strains were isolated from various sources, including 15 different host species (48 strains from humans) and environmental samples. Pathogenic bacterial strains identified from human clinical infections were also included in the study. As a result of whole genome database screening, a total of 80 heterogeneous AMR genes were identified from the whole-genome datasets of 99 *P. mirabilis* isolates (Additional file 3). PlasmidFinder 2.1 analysis revealed 3 types of plasmids, namely *Col3M*, *IncC* and *IncQ1*, identified in 6 *P. mirabilis* whole-genome datasets (Additional file 4). Of these, 5 strains carried *Col3M* (% identity 98.09) whereas *P. mirabilis* strain LB UEL H-11 (GenBank accession no. CP086377.1) carried *IncC* and *IncQ1*.

Whole chromosome SNPs were identified using the CSI Phylogeny pipeline, with *P. mirabilis* HI4320 (GenBank accession no. AM942759.1) as a reference gene for pairwise SNP difference measurement. The pairwise SNP difference (Additional file 5) ranged from 0 (between C74 and C55) to 45,353 (between MPE0156 and CCUG70746). The *P. mirabilis* strain XH1568 (GenBank accession no. CP049941.1) was revealed to have the smallest pairwise SNP difference (6302 positions) from LHPm1.

**Figure 3:**
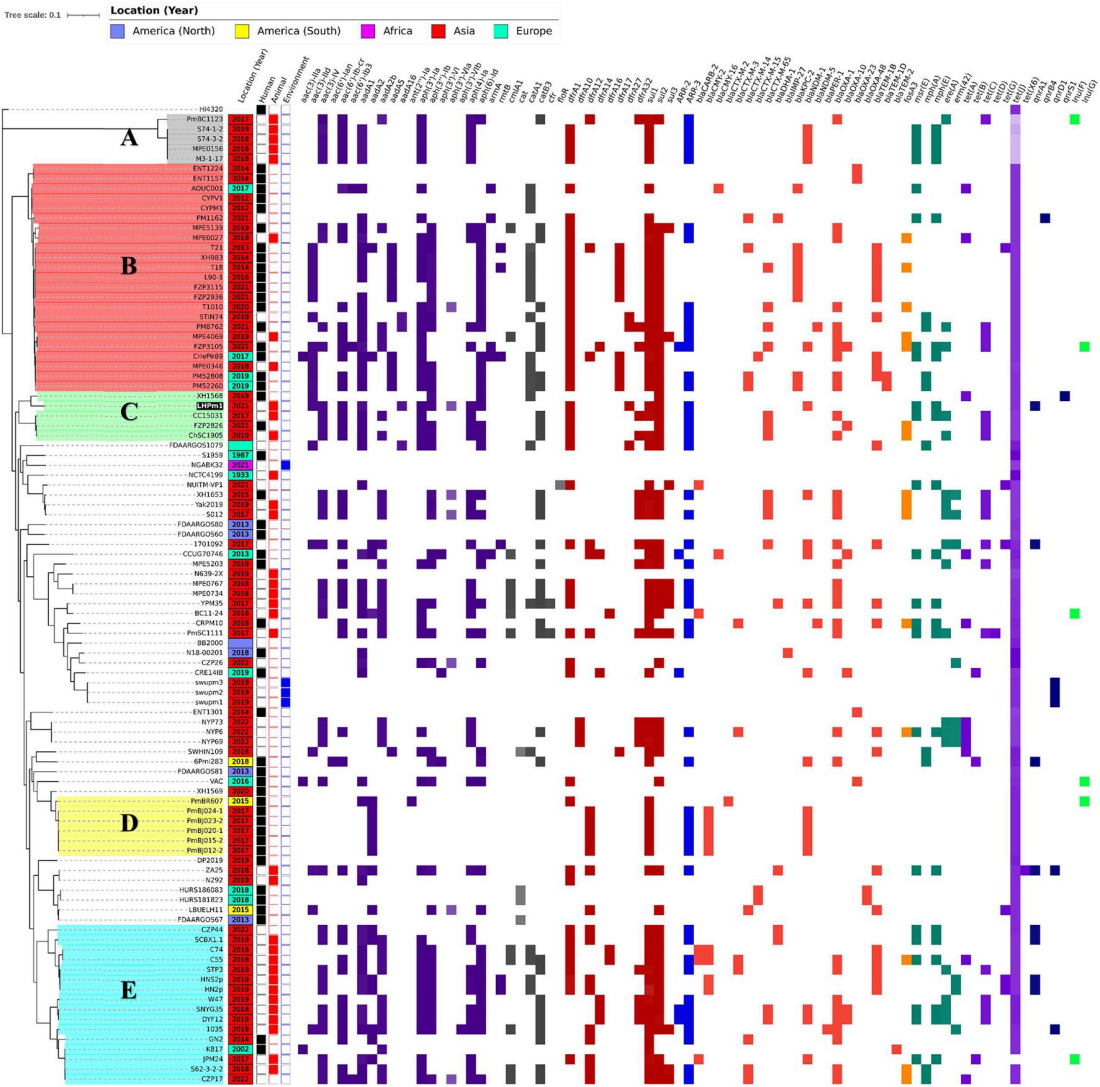
**Epidemiological comparison of the whole genome datasets of 99** ***P. mirabilis*** **strains.** The whole-genome SNP identification using *P. mirabilis* HI4320 (GenBank accession no. AM942759.1) as a reference was adjusted to construct a maximum likelihood (ML) tree and the results were visualized by iTOLs. The LHPm1 strain identified in this study is highlighted with a black background. The years that each strain was isolated are listed in the square boxes and colored differently according to their location of origin. The isolation source, whether from humans, animals, or the environment, is displayed using colored boxes. The AMR gene identities were displayed by heatmaps, colored differently according to the antimicrobial classes. The identified phylogroups (**A**–**E**) are depicted in distinguishable colored backgrounds.

Subsequently, a whole chromosome SNP-based ML tree was constructed based on the pairwise SNP difference. To gain a clearer epidemiological perspective of LHPm1, epidemiological information, such as isolative sources, location, year and the AMR gene identity heatmap, was visualized with the whole chromosome phylogenetic tree (Figure 3). As a result of the whole genome epidemiology of the *P. mirabilis* datasets, 5 phylogroups were distinguishable based on phylogenetic relatedness, isolative source or resistance gene distribution pattern. The identified phylogroups are depicted in Figure 3 with colored backgrounds. LHPm1 was grouped in phylogroup C (green background), which is a clade that can be characterized by highly distributed AMR genes.

## Discussion

In this study, carbapenemase NDM-1 carriage was revealed from the common rectal screen of companion animals, which are close members of human society yet outside of the major surveillance system of our community. Featuring high mortality and morbidity especially in nosocomial environments, most of the infections caused by MDR Enterobacterales are likely derived from the gastrointestinal (GI) tract [30, 31]. *P. mirabilis* is a commensal strain, and it has been suspected to be responsible for UTI infections originating from the GI tract [1, 2]. However, the host dog of the isolate in this study was healthy to the best of our knowledge, and showed no special symptoms or clinical situation. This is not strange because *P. mirabilis* is known as a commensal strain in the GI tract of humans and animals [1]. In human investigations of Korea, *P. mirabilis* was reported as the 5^th^ most prevalent (6.5%) urine isolate and 5^th^ most prevalent (2.8%) species among CREs [32–34]. Among carbapenemase-producing *P. mirabilis* isolates, the most prevalent carbapenemase type was NDM-1, consistent with the findings in this study. The NDM-1-producing *P. mirabilis* isolate in this study was the first strain detected in a dog in our country. In a nationwide surveillance study on dogs [35], *P. mirabilis* was reported as the 4^th^ most isolated species from stools (4.4%), 5^th^ from skin (3.7%), and 2^nd^ from urine (20.0%). There has been no clear report supporting direct dissemination or infection of *P. mirabilis* between humans and animals. However, considering the increasing role of companion dogs in human society, the discovery of NDM-1 harboring *P. mirabilis* in the GI tract of a companion dog should be taken seriously.

Although LHPm1 was isolated from a dog without any identified clinical symptoms, the clinical potential of MDR *P. mirabilis* as a pathogen should also be considered. Several key virulence factors of *P. mirabilis*, including iron acquisition systems, lipopolysaccharide, hemolysins, proteases, and flagella, are known to confer pathogenicity to the isolates [36, 37]. Although it is still unclear, swarming capability on surface is suspected to be correlated with urease and crystalline biofilm formation [38]. Fimbriae formation is also known to be responsible for adhering to the uroepithelium, localizing in the urinary tract, and forming biofilms [39, 40]. There are 17 important virulence factors reported to affect fimbriae formation, including the well-known mannose-resistant *Proteus*-like (MR/P) fimbria [41]. The genomic variations identified in this study (Figure 2) did not include any differences of known virulence factors of *P. mirabilis*. Interestingly, pathogenic factors such as swarming motility, mutual growth, and biofilm formation were reported to be correlated with the AMR capacity of *P. mirabilis* [42]. MDR *P. mirabilis* strains exhibited higher mutual growth and biofilm formation but lower swarming motility. However, LHPm1 was identified in a dog without any clinical complaints, and the clinical potential of the strain was not assessed in this study. Therefore, potential clinical impact of the LHPm1 should not be neglected, and further investigation into its potential pathogenicity should be undertaken.

The result of whole-genome resistance gene identification using a public database was consistent with the MIC values. The MIC evaluation revealed resistance to multiple classes of antibiotics, including β-lactam agents such as cephalosporins, penicillins and carbapenems, aminoglycosides, chloramphenicol, macrolides and polymyxins. NDM-1 is a well-known carbapenemase, that confers resistance against β-lactam agents by hydrolyzing antibiotics containing β-lactam rings [43]. The resistance level of LHPm1 against meropenem, cephalosporins (ceftazidime and ceftriaxone), and penicillin (amoxicillin) seems to be due to the presence of NDM-1. Various aminoglycoside genes were identified in the chromosome of LHPm1, namely *aac(3)-Iid, aac(3)-IV*, *aac(6’)-Ib-cr*, *aadA1*, *aadA16*, *aph(3’)-Ia, aph(3’’)-Ib*, *aph(3’)-*Via, *aph(4)-Ia* and *aph(6)-Id* [44]. These genes are speculated to be responsible for the resistance of LHPm1 to gentamicin. The loss of mobile gene elements in the genomic region (Figure 2), such as IS*CR1*, the *istAB* operon, and IS*1353*, indicates that the AMR genes linked with these genes would be stable in the chromosome of the LHPm1 [45]. The variations in the genomic region could stabilize the AMR genes and drug resistance ability derived from this region, such as aminoglycosides, rifampicin, and sulfonamides, potentially making treatment using these drugs difficult. The resistance of the isolate to doxycycline seems to be due to the presence of the *tet(J)*. The MIC determination also revealed the resistance of the isolate to chloramphenicol, which is believed to be due to the existence of the resistance genes *catB3* and *floR*. Similarly, *qnrA1* is suspected to be responsible for the resistance to nalidixic acid [46].

Tn*7*-like transposons contribute to the horizontal transfer of AMR genes among bacterial species via transposases [47]. However, the role of Tn*7*-like transposons in MDR isolates in our society remains largely unexplored. In this study, multiple genetic variations were discovered in LHPm1 and comparatively visualized with the Tn*7*-like structures Tn*6450* (GenBank accession no. MF805806.1) and Tn*6765* (GenBank accession no. MT503200.1).

The identification of MDR genes associated with multiple mobile genes from companion dogs is highly worrisome, considering the increasing role of animals in our society. Furthermore, MDR genes coupled with multiple mobile gene elements were identified with two tandem copies of *bla*_*NDM-1*_, which could confer an even broader spectrum of MDR capacity to the strain. Two tandem copies of *bla*_*NDM-1*_ have been reported from multiple species of bacterial chromosomes in previous reports [48–50], including *P. mirabilis*. Isolates with multiple copies of *bla*_*NDM-1*_ are known to show elevated carbapenem resistance [50]. In this study, 2 copies of *bla*_*NDM-1*_ were identified in *P. mirabilis*, yet one of them seems to have undergone a frame shift of the amino acid sequence. Although the other copy of *bla*_*NDM-1*_ seems to be enough to confer carbapenem resistance for the isolate (MIC value higher than 64 µg/mL), the underlying reason for the identified mutation remains unknown.

The whole-genome epidemiological study has reconfirmed that *P. mirabilis* is a ubiquitous bacterial species that can be isolated from humans, animals (both wildlife and domestic) and the environment. The whole-genome study revealed various classes of AMR genes in *P. mirabilis* datasets. Tetracycline resistance genes, mostly *tet(J)*, were identified in all of the investigated *P. mirabilis* strains. Among the datasets, the largest number of AMR genes was identified in FZP3105, with a total of 28 different AMR genes. Although only whole-genome datasets of significant isolates tend to be accessible in GenBank, it is clear that *P. mirabilis* is capable of carrying various AMR genes. In the whole genome phylogeny, 5 phylogroups were identified. Interestingly, isolates grouped in an identical phylogroup had a similar resistance gene distribution pattern. Isolates of phylogroup A originated from various animal species in China and carried either *bla*_*NDM-1*_ or *bla*_*OXA-1*_. Phylogroup B, C and E were characterized by a high distribution of aminoglycoside, amphenicol, and folate pathway antagonist resistance genes, relative to other phylogroups. Phylogroup D was identified with extended-spectrum β-lactamase (ESBL) and carbapenemase genes, yet other AMR genes, such as aminoglycoside, macrolide and quinolone resistance genes were relatively less prevalent. LHPm1 was included in phylogroup C, which is capable of colonizing both humans and animals, and was found to carry various AMR genes.

In phylogroup C, four *P. mirabilis* strains were closely grouped with LHPm1, raising concerns about serious clinical situations for humans. The four isolates of phylogroup C included two strains isolated from humans and the other two from animals. *P. mirabilis* strain XH1568 was identified from a gallbladder sample collected in China [51], from a patient diagnosed with gallbladder carcinoma. An ESBL *bla*_*CTX-M-65*_ producing isolate FZP2826 was also isolated in human patient [52]. *P. mirabilis* strain CC15031 was isolated from a canine diarrhea sample [53] and characterized to demonstrate high pathogenicity and antimicrobial resistance. An MDR *P. mirabilis* strain ChSC1905 was from nasal swab of a diseased pig of swine farm [54]. Given the host range of phylogroup C and its proximity to LHPm1, the potential for upcoming human infections must be taken seriously.

Therefore, novel control measures, from the perspective of the “one health approach”, are needed for public health, based on the findings from the whole-genome epidemiology.

This study describes the first carbapenemase-producing *P. mirabilis* strain isolated from a companion animal in South Korea. The identified whole-genome structure revealed 20 different AMR genes, including 2 tandem copies of *bla*_*NDM-1*_, which contributed to the MDR capacity of the isolate. Multiple variations in MDR gene regions and phylogenetic relatedness were identified by whole-genome analysis. The findings from the carbapenemase-producing *P. mirabilis* strain from a companion animal indicate that the problem of AMR is not limited to human health and should be addressed from the perspective of the “one health approach”.

### Supplementary Information

**Additional file1. The whole genome profiles of sequenced P. mirabilis LHPm1.**

**Additional file2. Epidemiological profiles of analyzed whole genome datasets in this study.**

**Additional file3. The dissemination metadata of antimicrobial resistance genes carriage in the whole genome datasets, searched by in silico screening by ResFinder 4.1 database.**

**Additional file4. Plasmid types metadata identified by PlasmidFinder 2.1 and presented as gene identity (%).**

**Additional file5. The pairwise SNP difference matrix of whole chromosome datasets extracted from the reference genome.**

## Data Availability

Publicly available datasets were analyzed in this study. This data can be found in Additional file 2 for all accession numbers.

## Abbreviations

AMR: Antimicrobial resistance
CGE: Center for Genomic Epidemiology
CLSI: Clinical and laboratory standards institute
CRE: carbapenem-resistant enterobacterales
ESBL: extended-spectrum β-lactamase
GI: gastrointestinal
ICEs: integrative conjugative elements
MALDI-TOF–MS: matrix-assisted laser desorption ionization–time of flight-mass spectrometry
MDR: multi-drug resistance
MIC: minimum inhibitory concentration
MIM: meropenem impregnated MacConkey
ML: maximum likelihood
NDM-1: New Delhi metallo-β-lactamase 1
NGS: next generation sequencing
SNP: single nucleotide polymorphism
TMP-SMX: trimethoprim-sulfamethoxazole
UTIs: urinary tract infections
