# Correction: Emergence and genomic characterization of *Proteus mirabilis* harboring *bla*_NDM-1_ in Korean companion dogs

**DOI:** 10.1186/s13567-024-01317-7

**Published:** 2024-04-29

**Authors:** Su Min Kyung, Jun Ho Lee, Eun‑Seo Lee, Xi‑Rui Xiang, Han Sang Yoo

**Affiliations:** Department of Infectious Disease, College of Veterinary Medicine, Seoul National University, Seoul, Republic of Korea

**Correction** **:** **Veterinary Research (2024) 55:50** 10.1186/s13567-024-01306-w

Following publication of the original article [1], we have been informed that there is a spelling in the title.

The incorrect title is: Emergence and genomic chion of *Proteus mirabilis* harboring *bla*_NDM-1_ in Korean companion dogs

The correct title is: Emergence and genomic characterization of *Proteus mirabilis* harboring *bla*_NDM-1_ in Korean companion dogs

The original article has been corrected.
