# Supplementary material for: Emergence and genomic characterization of Proteus mirabilis harboring blaNDM-1 in Korean companion dogs
Source: Vet Res. 2024 Apr 9;55:50. doi: 10.1186/s13567-024-01306-w (PMC11005143; doi:10.1186/s13567-024-01306-w)
Supplement: Supplementary file 1 — Additional file1. The whole genome profiles of sequenced P. mirabilis LHPm1. [file 13567_2024_1306_MOESM1_ESM.docx]

**Additional file 1 The whole genome profiles of sequenced *P. mirabilis* LHPm1.**

| **Strain** | **Contigs** | **Contig max length** | **N50** | **Contig total length** | **Total reads** | **Mapped reads** | **Mapping rate (%)** |
| --- | --- | --- | --- | --- | --- | --- | --- |
| **LHPm1** | 1 | 4 000 428 | 4 000 428 | 4 000 428 | 61 764 154 | 61 440 242 | 99.48 |

The whole genome records of sequenced *P. mirabilis* LHPm1. Long read genomic sequencing with Oxford Nanopore (Oxford Nanopore Technologies, Oxford, UK) platforms was corrected using Illumina NovaSeq 6000 (Illumina, San Diego, CA, USA) following a paired-end 2 × 150-bp protocol. Summarized alignment and assembly results is listed to confirm the reliable high quality of acquired whole genome sequence.
